# A novel assessment of adolescent mobility: a pilot study

**DOI:** 10.1186/s12966-015-0176-6

**Published:** 2015-02-15

**Authors:** Tom Stewart, Scott Duncan, Basile Chaix, Yan Kestens, Jasper Schipperijn, Grant Schofield

**Affiliations:** Human Potential Centre, Auckland University of Technology, Private Bag 92006, Auckland, 1142 Auckland New Zealand; Inserm, U707, Paris, France; CRCHUM, Department of social and preventive medicine, Université de Montréal, Montreal, Canada; Research Unit for Active Living, Department of Sport Science and Clinical Biomechanics, University of Southern Denmark, Odense, Denmark

**Keywords:** Active travel, Adolescent, Built environment, Mobility, Neighbourhood definition, Physical activity, Spatial polygamy, VERITAS, SoftGIS

## Abstract

**Background:**

The accurate measurement of daily mobility and travel to destinations beyond the residential neighbourhood has been identified as an important but almost systematically overlooked factor when investigating the relationship between exposure to the built environment and physical activity. The recent development of VERITAS – a web-based application nested within a computer-assisted personal interview – allows researchers to assess daily mobility, travel to regular destinations, and perceived neighbourhood boundaries using interactive mapping technology. The aims of this pilot study were to (1) demonstrate the feasibility and functionality of using VERITAS in an adolescent sample, and (2) compare urban form characteristics and geometric features of the perceived neighbourhood with traditional neighbourhood delimitations.

**Methods:**

Data were collected and analysed for twenty-eight participants (14 male, 15.9 ± 1.48 years) in 2013. Participants underwent anthropometric assessment before completing a custom-designed VERITAS protocol under the supervision of trained interview technicians. Regularly visited destinations, school travel routes, transportation modes, travel companions, and perceived neighbourhood boundaries were assessed. Data were imported into ArcGIS and street network distances between the home and each geolocated destination were generated. Convex hull activity spaces were derived from destinations. Urban form variables and geometric characteristics were compared between the perceived neighbourhood, existing meshblocks, 1 mile Euclidean buffers, and 1 km network buffers.

**Results:**

In total, 529 destinations were geolocated, 58% of which were outside the perceived neighbourhood boundary. Active travel was inversely associated with distance to destinations (*r* = −.43, *p* < .05) and traveling with adults (*r* = −.68, *p* < .01). Urban form and geometric characteristics of the perceived neighbourhood were different from those in other neighbourhood delimitations.

**Conclusions:**

This study demonstrates the feasibility of using VERITAS to assess mobility within adolescent populations. Our results also illustrate the potential novelty and use of user-defined spaces, and highlight the limitations of relying on restricted definitions of place (i.e., administrative or residential-focused neighbourhoods) when assessing environmental exposure.

## Background

Physical inactivity is a key contributor to the widespread prevalence of non-communicable disease [1]. Behavioural and motivational approaches to increase physical activity have had relatively limited effectiveness [2], causing researchers to consider how environmental and policy factors may affect behaviour and health [3]. An accurate assessment of environmental exposure is paramount for the clarification of these relationships and the development of supportive policy. The environment contiguous to the principal residence is unquestionably important when investigating interactions with the environment, but current methods of neighbourhood delimitation are equivocal [4], and the residential neighbourhood is not the sole mechanism that links place to health [5].

Previous health studies have estimated environmental exposure using predefined administrative area subdivisions [6,7], ego-centred definitions of space; such as buffers of varying distances and types around the principal residence [4], and mental maps [8,9]. However, the environment to which individuals are exposed may differ substantially from these ‘residential neighbourhood’ type measures: individuals are not normally confined within these spatial boundaries, and visit an array of destinations beyond the perimeter of their residential neighbourhoods (known as spatial polygamy) [10]. Having focussed exclusively on residential neighbourhoods as an area delimitation to assess environmental exposures, the majority of these studies have succumb to the ‘local trap‘ [11], and overlooked the concept of spatial polygamy, resulting in a potential mischaracterisation of environmental exposure [12].

The everyday movement of individuals over space between destinations, known as daily mobility, has been recognised as an important component that needs to be accounted for in the assessment of environmental exposure [13,14]. Mobility is also important for identifying the shape and scale of exposure, which may vary between different population groups. For example, the proportion of daily mobility trajectories inside and outside the neighbourhood may differ between adults and youth or adolescent groups who interact more with their local resources and infrastructure. Mobility is not only important to enhance the assessment of environmental exposure, access to resources, and feelings of neighbourhood belonging [15], but also as a potential source of active transport [16]. A recent study showed adolescents living in urban areas accumulated 57% of moderate-to-vigorous physical activity (MVPA) while commuting to activity places, rather than at the destinations themselves [17].

Activity spaces have been proposed to characterise the spatial patterns of mobility [5,18]. Activity spaces are expressions of spatial behaviour which enclose the principal residence, the destinations where individuals spend their time, and the travel routes between these destinations [19]. These measures are thought to be more comprehensive spatial summaries of mobility and experienced spaces compared to traditional neighbourhood measures [5]. Activity spaces are commonly derived from convex hulls [20], standard deviational ellipses [21] or travel time polygons [18] and likely encompass environments both inside and outside the residential neighbourhood, yet only 4% of studies in a recent review investigated both [13]. The few studies that have included locations outside the neighbourhood have mainly focused on fixed spatial daily life centres, such as the workplace or school, yet minor activity locations and the travel between them are also of interest [5]. Significant differences between environmental characteristics within the neighbourhood and beyond the neighbourhood have been shown [22] justifying the use of activity spaces in addition to residential neighbourhood measures.

Early evidence for daily mobility was primarily collected using retrospective mobility surveys [23], and later real-time travel diaries [24] where participants were asked to keep detailed accounts of all trips made. However, detailed information requires a high level of participant engagement and accuracy, which increases participant burden and can lead to incomplete and incorrect information [25]. Even though these data can be used to estimate trip lengths, frequencies, and travel modes, there is an absence of the exact itineraries followed [26]. With technological advances, the use of portable Global Positioning System (GPS) receivers to measure outdoor movement is becoming a more feasible and cost-effective solution [27]. Although GPS receivers can obtain a comprehensive record of spatiotemporally referenced data, GPS measurement is still somewhat hindered by technological constraints, such as signal dropout, memory limitations and poor battery life [28]. Furthermore, the cleaning and processing of GPS data requires significant time and expertise [29], which may limit the size and scale of GPS studies, although improved methods are rapidly reducing the significance of these problems [30]. More recently, electronic activity location questionnaires with integrated interactive mapping capabilities have been proposed to enhance the geographic accuracy of data, ease of collection, and the possibility of collecting additional information such as perceived spaces or limits of independent mobility [16]. Such approaches offer a practical alternative for mobility assessment, and have shown high convergent validity when compared with GPS travel records [20,31].

The Visualization and Evaluation of Route Itineraries, Travel Destinations, and Activity Spaces (VERITAS) is a web-based application delivered within a Computer-Assisted Personal Interview (CAPI) that integrates interactive mapping functionality (based on Google Maps) with traditional activity and travel questions [16]. This enables participants to accurately geolocate regular destinations inside and outside of the residential neighbourhood, answer questions related to each of those destinations, and draw lines and polygons indicative of routes and spaces. VERITAS can assess daily mobility over extended retrospective periods, which may provide more relevant comparisons with chronic health indicators such as BMI. VERITAS was initially proposed and developed for the RECORD Study – a longitudinal study focusing on the links between the environment and health in French adults [32] – and has yet to be trialled within an adolescent sample.

The aims of this pilot study were to (1) demonstrate the feasibility and functionality of using VERITAS in an adolescent sample, and (2) compare urban form characteristics and geometric features of the perceived neighbourhood with traditional neighbourhood delimitations. It is hoped this information will contribute to the next generation of built environment and mobility studies, and help elucidate the links between the built environment, physical activity, and health.

## Methods

### Participants

Twenty eight adolescent participants (13 – 18 years) were recruited from an Auckland high school as a subsample of participants in the Built Environment and Adolescent New Zealanders (BEANZ) study – a cross-sectional study exploring the links between the built environment and health in New Zealand adolescents. The BEANZ recruitment procedures are described in detail elsewhere [33]. Briefly, New Zealand meshblock (smallest census tract unit) walkability indices were calculated for all eligible participants based on their residential addresses, which were obtained from the school’s database prior to the consent process. The walkability indices used were consistent with previous research in New Zealand adults [34]. The subsample was selected from the pool of consenting students, with half of the sample randomly selected from the lowest walkability tertile and the remaining half from the highest tertile in an attempt to achieve variation in environmental exposure [34]. Ethical approval was granted by the Auckland University of Technology Ethics Committee (AUTEC), and written informed consent was obtained from each student and parent prior to participation.

### Instruments

**Figure 1:**
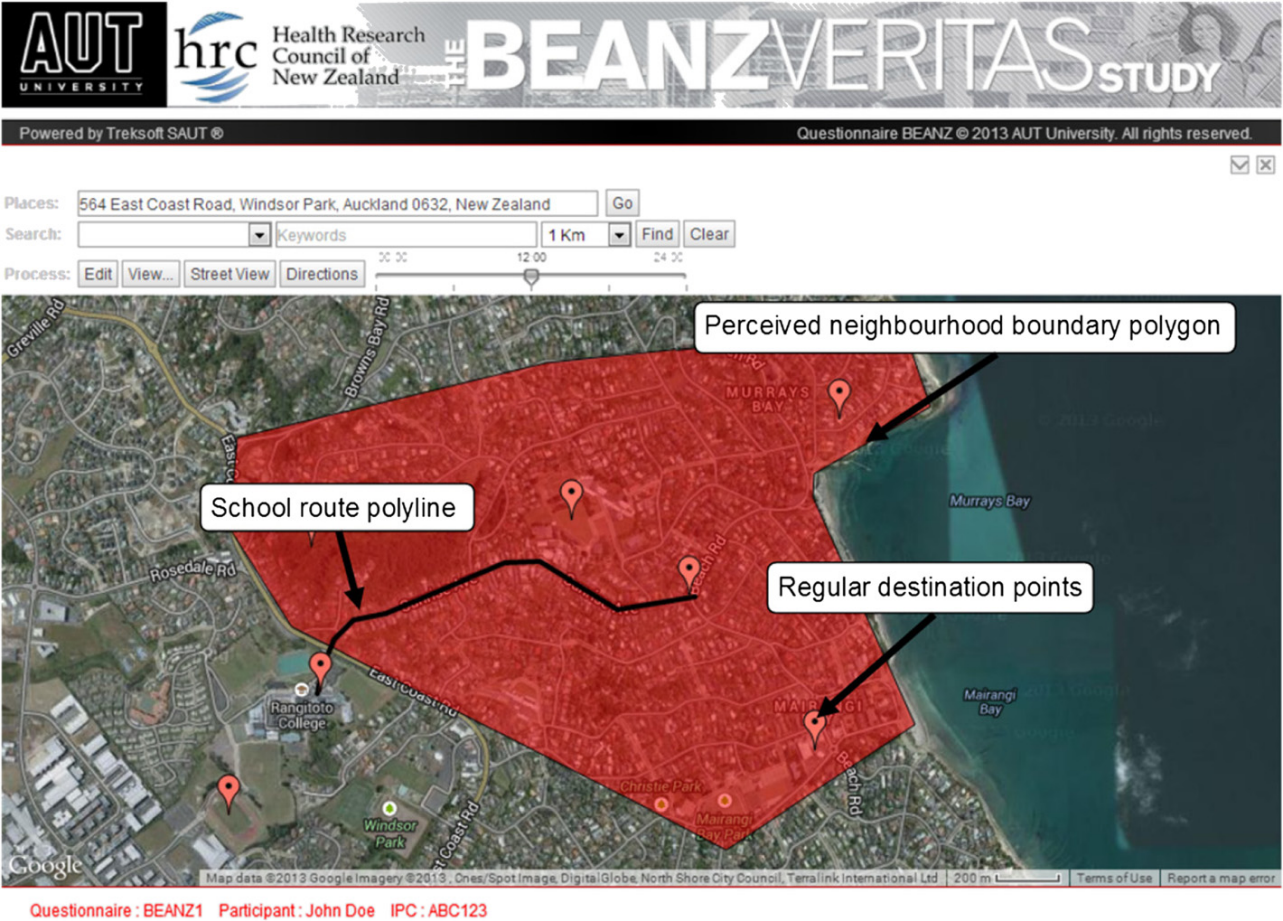
**Google Maps tools embedded within VERITAS-BEANZ.**

VERITAS-BEANZ was developed by translating VERITAS-RECORD from French to English. The conception of VERITAS-RECORD is described in more detail elsewhere [16]. Elements of the NEWS-Y [35] questionnaire were incorporated and adjusted to suit the New Zealand adolescent sample (i.e., including netball and rugby league as sporting options, and push scooters as a mode of travel option). VERITAS-BEANZ has five successive parts: (1) locating the principal residence (and secondary residence if necessary), answering questions about its occupants, and recording the level of neighbourhood attachment on a 1–6 Likert scale, (2) selecting the types of places visited in the previous six months from a list of destination categories (e.g., school, bank, post office), (3) geolocating the most frequently visited destination within each of the selected destination categories (e.g., the post office visited most frequently), and answering questions related to that destination; such as visit frequency, mode of travel, travel companions, and whether they are allowed to go there without adult supervision, (4) plotting the usual route travelled to school (and from school if necessary) by placing a series of points which connect to form a polyline (Figure 1), and (5) plotting their perceived neighbourhood boundary by placing a series of points, which connect to form a polygon (Figure 1).

Unlike VERITAS-RECORD, plotting the perceived neighbourhood boundary occurred after geolocating regular destinations. It is possible that the dispersal of previously located destination markers may influence a participant’s perception of their neighbourhood, yet this order of steps was selected in hopes it would improve the accuracy of the boundary delimitation. Before plotting their perceived neighbourhood, their principle residence was positioned at the centre of the map. Participants were then asked to “draw a shape which you feel represents your neighbourhood” and were ensured there were no right or wrong answers [36].

Participants identified frequently visited destinations from a list of 33 categories extracted from the NEWS-Y questionnaire, as well as dedicated questions for sport, cultural and religious activities, and visiting friends. To assist with destination identification, VERITAS-BEANZ is equipped with Google Street View and embedded search tools which can identify destinations of a particular type (or from key search terms) within a given radius (Figure 1). Altogether, VERITAS-BEANZ contains a maximum of 188 individual questions (some of which may have multiple answers); although participants do not have to answer questions related to destinations they have not visited.

### Procedure

Data collection took place at an Auckland high school in June 2013 over a 3 day period. Height was measured to the nearest 0.1 cm using a portable stadiometer (SECA 213, Hamberg, Germany), weight to the nearest 0.1 kg using electronic scales (SECA 813, Hamberg, Germany) and waist circumference at the navel to the nearest 0.1 cm using a Lufkin Executive Thinline steel tape measure (W606PM, Cooper Hand Tools, NC, USA) in line with ISAK-developed protocols [37]. Trained interview technicians took each participant through VERITAS-BEANZ on a laptop computer which was connected to the school’s wireless local area network (Wi-Fi). During the interview, any questions or aspects of VERITAS which participants had trouble understanding were made note of, and discussed with the research team at the conclusion of each day’s data collection session. Answers and map data were automatically saved to our dedicated server during and at completion of the interview.

### Data reduction

VERITAS-BEANZ questionnaire data (mode of travel, frequency of visits, travel companions) and map data (destination, polyline, and polygon coordinates) were downloaded and imported into ArcGIS 10.1 (ESRI, Redlands, CA, USA). Perceived neighbourhood boundary coordinates were then converted to polygons using the ET GeoWizards (ET Spatial Techniques, Faerie Glen, Pretoria) point to polygon conversion tool. The shortest network route between the principal residence and each mapped destination was estimated using the Network Analyst Extension and street centreline data obtained from the Land Information New Zealand (LINZ) database (www.linz.govt.nz). Using VERITAS-BEANZ questionnaire data, each of these estimated travel routes were coded as either active travel (i.e., walking, cycling), passive travel (i.e., motorised transport) or mixed travel, which was defined as a combination of both passive and active travel (whether individual trips were multimodal, or different travel modes were used for different trips). All frequency of visits data (reported as either times per week, month, or year) were all converted to times per year for comparative purposes. Using the distance, frequency and mode of travel, a weighted distance metric was computed to estimate the annual distance accumulated by each mode of travel, whilst travelling to each destination.

**Figure 2:**
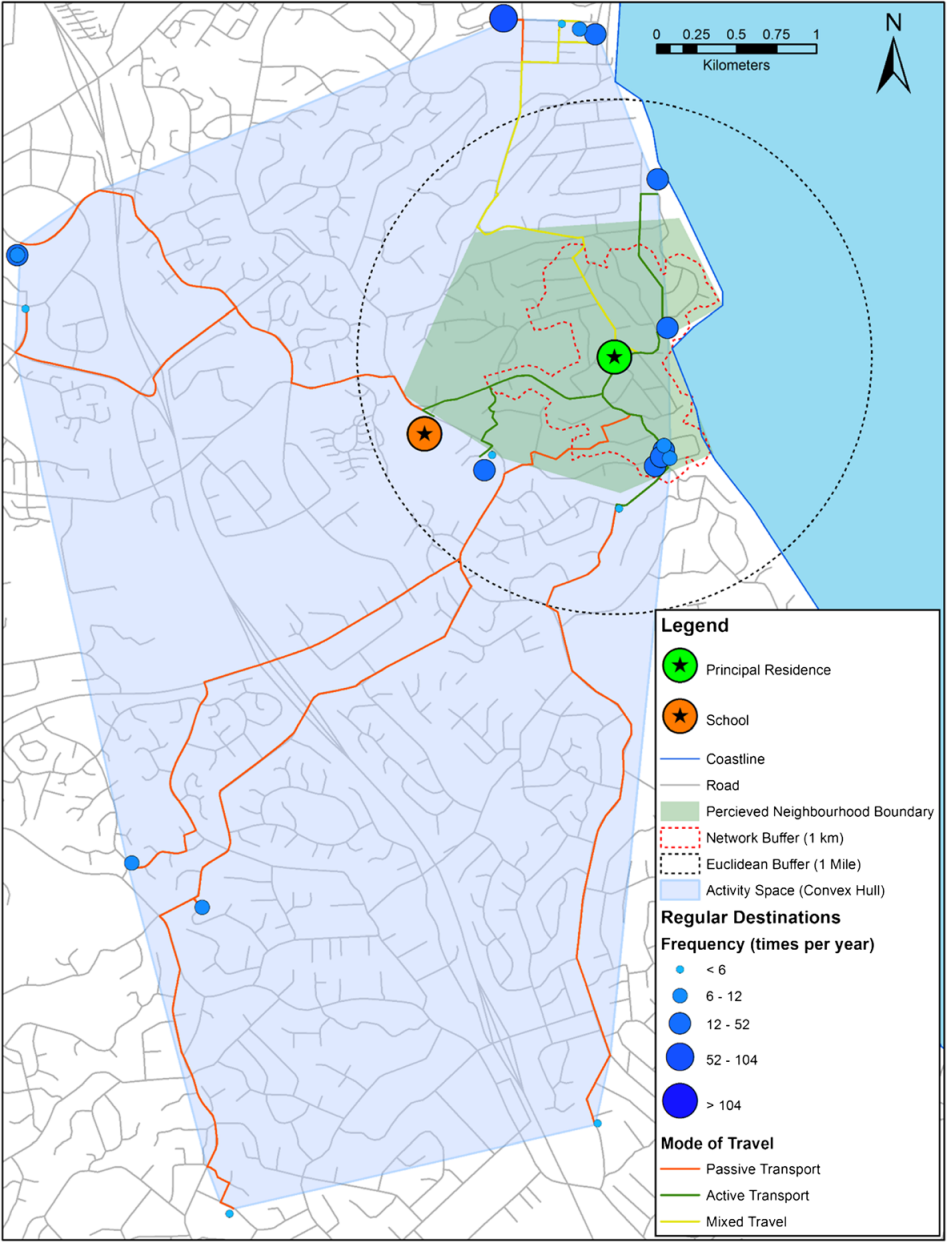
**A single participant’s VERITAS data presented in ArcGIS 10.1.**

1 mile Euclidian buffers, 1 km network buffers and corresponding meshblocks were generated for the purpose of comparing the perceived neighbourhood. These buffer distances were chosen because they have commonly been used in adolescent samples [38-42]. A convex hull is a minimum bounding geometry technique which encloses multiple geographic features within the smallest possible convex polygon [20], and was used to define activity space by enclosing all geolocated destinations. Excluding destinations which are visited rarely may provide more representative spatial summaries of typical travel behaviour, but due to the pilot nature of this study, all destinations that were located during the BEANZ-VERITAS questionnaire were included in the activity space delineation (Figure 2). The ArcGIS XY to line and Generate Near Table tools were used to calculate the distance from the principal residence to the farthest boundary vertex, and the distance to the closest edge of each neighbourhood delimitation and activity space. The uniformity of each neighbourhood polygon around the principal residence was assessed using the ratios of these two distances along with shape circularity. Circularity is a measure of how closely a shape resembles a circle, and is defined as the ratio of the area of a shape with the area of a circle which has the same perimeter [43]. Circularity was calculated using the equation:

$$ Circularity=4\pi A/P\hat{\mkern6mu} 2 $$

where *A* is the area and *P* is the perimeter of the shape. The circularity ratio ranges from 0 to 1, the latter indicating a perfect circle [44]. For each neighbourhood delimitation, the percentage of area that overlapped the perceived neighbourhood was also calculated.

### Measures of urban form

Urban form variables have been inconsistently associated with adolescent physical activity in the past [45], although the imprecise assessment of the residential neighbourhood may have contributed to these discrepancies [4]. Three distinct measures of urban form (land use mix, street connectivity, and residential density) were calculated within each of the neighbourhood delimitations. Auckland Council zoning data were used to calculate land use mix. Land was categorised as residential, commercial, industrial, open space, or other. Entropy scores were used to calculate the extent of land use mix using the equation:

$$ Land\kern0.5em  Use\kern0.5em  Mix=-1\left({\displaystyle {\sum}_{i=1}^n{P}_i}* \ln \left({P}_i\right)\right)/ \ln (n) $$

where *n* is the number of different land use categories and *Pi* is the proportion of land use category *i* in the region. Entropy scores range from 0, which indicates no mix or homogeneous land use, to 1 which represents heterogeneous land use, or a perfect mix. Street connectivity was estimated by calculating the number of intersections with three or more intersecting streets per square kilometre. Intersections were extracted from pedestrian road network data (i.e., with non-walkable elements such as highways and on/off ramps removed). All intersections within 10 m were considered one intersection to account for roads that may not align perfectly. As meshblock boundaries are normally defined by street centrelines, a 20 m buffer was applied to each meshblock to include peripheral intersections which may otherwise be omitted. The number of private dwellings per meshblock was obtained from the New Zealand 2006 census [46]. Residential density for each meshblock was calculated by dividing the total number of private occupied dwellings by meshblock area. Residential density was calculated within each polygon and buffer by estimating the number of private dwellings using an area weighted average based on meshblock-level data. The handling of these urban measures was consistent with previous studies [34].

### Analysis

Descriptive statistics (mean ± SD) were generated for all data. Normality tests revealed non-normally distributed data so nonparametric analyses were performed. Wilcoxon signed rank tests were used to assess differences between residential neighbourhood delimitations, Mann–Whitney U tests were used to assess differences between genders, and Spearman’s correlations were used to test for associations between trip distances, travel modes and travel companions. Significance was set at *p* < .05, and all analyses were conducted using IBM SPSS Statistics v22 (IBM Cooperation, USA).

## Results

The demographic characteristics of the sample are presented in Table 1, and a summary of VERITAS-BEANZ statistics are presented in Table 2. In total, 529 individual destinations were geolocated (mean = 17.9 ± 5.11), with similar numbers between genders. The number of destinations that participants had visited but were unable to locate was 76, although 36.8% of these were from three participants who were unfamiliar with interpreting maps. The time taken to complete the questionnaire averaged 28.3 ± 9.4 minutes, and was significantly correlated with the number of destinations geolocated (*r* = .61, *p* < .01). Overall, 41% of visited destinations were inside the perceived neighbourhood boundary, with females showing a slightly higher percentage than males (44.73 and 38.54%, respectively), although this difference was not significant (*p* = .56). The level of neighbourhood attachment (mean = 4.39 ± 1.13) was unrelated to the number of destinations that fell inside the perceived neighbourhood (*r* = .11, *p* = .57) or its area (*r* = −.08, *p* = .70).

**Table 1:** **Participant demographic characteristics (mean ± SD)**

	**Male n = 14**	**Female n = 14**	**All n = 28**
Age	15.84 ± 1.49	15.91 ± 1.53	15.88 ± 1.48
Height (cm)	174.24 ± 7.87	164.55 ± 4.78	169.40 ± 8.07
Weight (kg)	61.91 ± 9.80	57.45 ± 8.13	59.68 ± 9.12
BMI	20.33 ± 2.48	21.17 ± 2.42	20.75 ± 2.44
Waist circumference (cm)	72.58 ± 7.09	67.56 ± 5.12	70.07 ± 6.58

**Table 2:** **VERITAS statistics**

	**Male**	**Female**	**All**
	***n*** **= 14**	***n*** **= 14**	***n*** **= 28**
	**mean ± SD (min - max)**	**mean ± SD (min - max)**	**mean ± SD**
Time to complete VERITAS (min)	30.73 ± 11.61 (13.25 - 64.8)	25.79 ± 6.10 (16.8 - 42.75)	28.26 ± 9.44
Number of destinations geolocated	17.50 ± 5.5 (8–27)	18.29 ± 4.86 (13–29)	17.89 ± 5.11
Destinations inside perceived neighbourhood (%)	38.54 ± 31.18 (0 – 90)	44.73 ± 23.37 (0 – 81)	41.64 ± 27.22
Neighbourhood attachment (1 to 6 scale)	4.21 ± 1.25 (2–6)	4.57 ± 1.02 (2–6)	4.39 ± 1.13
Number of people living at address	4.43 ± 1.16 (3–7)	4.00 ± 1.30 (2–6)	4.21 ± 1.23

Table 3 shows the characteristics of each type of destination. On average, the closest destinations to home were public parks (0.89 ± 0.88 km), public transit stops (1.37 ± 2.68 km), schools with recreation facilities (1.47 ± 1.17 km), and convenience stores (1.48 ± 1.01 km). These four destinations also had the highest proportion of active transport trips (88.2, 81, 54.5, and 56% respectively). Overall, network distance to destinations was positively associated with passive travel (*r* = .68, *p* < .01), negatively associated with active transport (*r* = −.63, *p* < .01), and negatively associated with traveling alone (*r* = −.46, *p* < .05). The distance accumulated per year metric shows the relative importance of each destination for each mode of travel (although it assumes trips are made from the principal residence and thus ignores trip chains). Across the whole sample, the most active (457 km), passive (1067 km), and mixed (611 km) transport distance was accumulated during the commute to and from school. Passive transport distance was also high when travelling to organised sport (669 km) and indoor recreation facilities (634 km), whereas active transport distance was high when travelling to playing fields (291 km), public transit stops (257 km), and friends’ houses (167 km). Public open spaces and cultural or religious activities also had high active transport distances, although only one third of participants visited these types of destinations. Being allowed to travel to a destination without adult supervision was positively associated with traveling alone (*r* = .61, *p* = .01) and having friends as travel companions (*r* = .64, *p* < .01), but negatively associated with passive travel (*r* = −.50, *p* < .01). Active travel was positively associated with traveling with friends (*r* = .53, *p* < .01), siblings (*r* = .43, *p* < .05) and alone (*r* = .55, *p* < .01), but negatively associated with adults (*r* = −.71, *p* < .01).

**Table 3:** **Geolocated destination characteristics**

**Destination**	**Network distance (km)**	**Visited (%)**	**Frequency (times/year)**	**Mode of travel (%)**			**Weighted distance (km/year)** ^**a**^			**Without adults (%)**	**Travel companions (%)**			
				**Active**	**Passive**	**Mixed**	**Active**	**Passive**	**Mixed**		**Friends**	**Siblings**	**Parent**	**Alone**
Own School	4.09 ± 3.84	100	261 ± 10	21.4	50	28.6	456.89	1067.49	610.6	96.4	71.4	42.9	14.3	32.1
Playing Fields & Courts	3.59 ± 5.94	50	81 ± 57	50	50	0	290.79	290.79	0	100	57.1	28.6	42.9	42.9
Public Open Space	9.49 ± 8.47	32.1	32 ± 49	44.4	33.3	22.2	269.67	202.25	134.83	88.9	77.8	44.4	44.4	0
Public Transit Stop	1.37 ± 2.68	75	116 ± 109	81	14.3	4.8	257.45	45.45	15.26	100	57.1	33.3	9.5	57.1
Cultural or Religious	3.58 ± 2.78	35.7	113 ± 111	30	60	10	242.72	485.45	80.91	-	50	20	40	50
Visiting friends	1.76 ± 2.31	82.1	91 ± 70	52.2	26.1	26.1	167.21	83.6	83.6	-	30.4	21.7	26.1	65.2
Beach or Lake	3.63 ± 5.9	75	45 ± 35	47.6	33.3	19	155.51	108.79	62.07	95.2	81	33.3	66.7	33.3
Swimming Pool	6.84 ± 5.87	39.3	42 ± 49	18.2	72.7	9.1	104.57	417.71	52.28	81.8	27.3	45.5	72.7	9.1
Sport	4.93 ± 6.17	82.1	120 ± 68	8.7	56.5	34.8	102.94	668.51	411.75	-	56.5	30.4	52.2	21.7
Indoor Recreation	4.33 ± 4.19	57.1	90 ± 60	12.5	81.3	6.3	97.43	633.65	49.1	81.3	25	18.8	37.5	31.3
Basketball Court	2.41 ± 2.95	26.8	50 ± 37	37.5	50	12.5	90.38	120.5	30.13	87.5	75	62.5	25	25
School with Recreation Facilities	1.47 ± 1.17	39.3	56 ± 52	54.5	36.4	9.1	89.73	59.93	14.98	100	90.9	54.5	0	18.2
Public Park	0.89 ± 0.88	60.7	49 ± 32	88.2	0	11.8	76.93	0	10.29	100	76.5	35.3	29.4	35.3
Convenience Store	1.48 ± 1.01	89.3	42 ± 33	56	28	16	69.62	34.81	19.89	96	48	36	24	48
Any Other School	1.97 ± 1.8	39.3	26 ± 36	50	28.6	21.4	51.22	29.3	21.92	100	64.3	42.9	35.7	35.7
Fast Food	4.17 ± 5	82.1	31 ± 31	17.4	60.9	21.7	44.99	157.45	56.1	82.6	52.2	34.8	65.2	13
Walking Trails	2.29 ± 2.18	32.1	19 ± 33	44.4	55.6	0	38.64	48.38	0	55.6	33.3	55.6	88.9	11.1
Café	4.59 ± 4.41	78.6	24 ± 24	9.1	63.6	27.3	20.05	140.12	60.15	95.5	54.5	31.8	63.6	4.5
Video or DVD Store	2.05 ± 1.29	71.4	32 ± 24	15	40	45	19.68	52.48	59.04	100	55	35	75	25
Supermarket	2.29 ± 1.29	96.4	57 ± 35	7.4	59.3	33.3	19.32	154.81	86.93	92.6	18.5	37	81.5	7.4
Chemist	1.84 ± 1.43	67.9	22 ± 27	21.1	47.4	31.6	17.08	38.38	25.58	63.2	5.3	21.1	89.5	15.8
Bank	2.88 ± 1.52	82.1	18 ± 13	8.7	65.2	21.6	9.02	67.6	22.39	69.6	4.3	13	87	30.4
Restaurant	4.18 ± 4.76	71.4	17 ± 11	5	75	20	7.11	106.59	28.42	65	40	40	95	0
Post Office	2.85 ± 1.94	46.4	11 ± 9	7.7	69.2	23.1	4.83	43.39	14.48	69.2	0	23.1	84.6	15.4
Clothing Store	5.42 ± 3.22	82.1	36 ± 30	0	87	13	0	339.51	50.73	95.7	73.9	30.4	69.6	26.1
Bookstore	4.48 ± 2.82	39.3	30 ± 60	0	81.8	18.2	0	219.88	48.92	100	27.3	27.3	100	18.2
Library	4.39 ± 2.89	64.3	12 ± 8	0	77.8	22.2	0	81.97	23.39	94.4	44.4	16.7	55.6	27.8
Laundry	3.85 ± 0	3.6	1 ± 0	0	100	0	0	7.7	0	0	0	0	100	0

^a^Weighted distance = ((network distance × 2) × frequency) × (mode of travel ÷ 100).

Table 4 compares urban form characteristics and geometric features assessed within each neighbourhood delimitation. On average, the perceived neighbourhood boundary area (3.54 ± 2.64 km^2^) was larger than a NZ meshblock (0.12 ± 0.16 km^2^) and a 1 km network buffer (1.03 ± 0.33 km^2^) but smaller than a 1 mile Euclidean buffer (8.14 ± 0 km^2^). The proportion of area which fell inside the perceived neighbourhood boundary was highest in meshblocks (85.1%) followed by the 1 km network (79.4%) and 1 mile Euclidean buffers (34.6%). The distance to the farthest boundary vertex was considerably longer than the distance to the closest edge, and the ratios between these distances were similar in all neighbourhood delimitations apart from the 1 mile Euclidean buffer (which has perfect shape uniformity). The perceived neighbourhood boundary showed greater circularity (0.69 ± 0.15) than meshblocks (0.53 ± 0.15) and 1 km network buffers (0.0 ± 0.0). The urban form characteristics varied substantially between each neighbourhood delimitation, although residential density and street connectivity were similar between the 1 km network buffer and the perceived neighbourhood.

**Table 4:** **Neighbourhood and activity space geometry and urban form comparison (mean ± SD)**

	**VERITAS perceived neighbourhood**	**NZ meshblock**	**1mile Euclidian buffer**	**1 km network buffer**	**Activity space (convex hull)**
Area (km^2^)	3.54 ± 2.64	0.12 ± 0.16**	8.14 ± 0	1.03 ± 0.33**	19.67 ± 24.83
Perimeter (km)	7.38 ± 3.38	1.57 ± 0.90**	10.11 ± 0	5.98 ± 1.45**	19.56 ± 10.89
Home to farthest boundary edge (km)	2.44 ± 1.38	0.53 ± 0.37**	1.61 ± 0	0.9 ± 0.09**	6.77 ± 4.3
Home to closest boundary edge (km)	0.54 ± 0.39	0.05 ± 0.04**	1.61 ± 0	0.18 ± 0.11**	0.36 ± 0.31
Farthest to closest edge ratio	10.88 ± 13.33	14.44 ± 12.55	1 ± 0**	13.48 ± 27.24	26.06 ± 24.53
Neighbourhood circularity	0.69 ± 0.15	0.53 ± 0.15**	1 ± 0**	0 ± 0**	0 ± 0
Perceived neighbourhood overlap (% area)^a^	-	85.12 ± 27.39	34.64 ± 21.73	79.43 ± 27.11	25.16 ± 23.54
Land Use Mix (entropy score)	0.38 ± 0.21	0.22 ± 0.16*	0.47 ± 0.15*	0.29 ± 0.12**	0.60 ± 0.16
Residential Density (dwellings/km^2^)	807.83 ± 233.75	894.17 ± 321.34**	627.34 ± 180.73**	854.29 ± 199.47	674.31 ± 189.0
Connectivity (intersections/km^2^)	45.35 ± 17.93	23.71 ± 23.7**	59.89 ± 21.18**	40.99 ± 7.7	52.01 ± 11.41

*Note*: *Significantly different from VERITAS perceived neighbourhood (*p* < .05).

**Significantly different from VERITAS perceived neighbourhood (*p* < .01).

^a^Calculated as the percentage of each neighbourhood’s area that fell inside the perceived neighbourhood boundary.

## Discussion

Although the built environment has been shown to contribute to young people’s activity and health behaviours [3,47], current evidence is inconsistent and exactly how the environment exerts its influence remains largely unclear [45,48]. As such, daily mobility and spatial polygamy have been identified as important elements to further our understanding of the environment-health relationship [5,10]. The development of electronic activity destination questionnaires which incorporate interactive mapping technologies are thought to allow a more comprehensive assessment of daily mobility and perceived spaces. This study sought to demonstrate VERITAS-BEANZ as an innovative method to assess mobility in adolescents.

Our results indicate that locating destinations of interest on an electronic map were relatively straightforward for the majority of participants regardless of gender and age. The interview technician’s local area knowledge proved to be beneficial when helping participants locate destinations, especially individuals who were not accustomed to reading maps. The search tools embedded within VERITAS were useful when participants knew the name of the destination and an approximate location, but were unable to pinpoint the destination itself. It was found that displaying the map using a hybrid view (i.e., a satellite view superimposed with roads and street names) assisted participants in pointing out familiar landmarks (such as treed areas and clusters of buildings) that were not visible on a simple street map (Figure 1).

Table 3 shows that certain destinations feature more regularly within adolescent’s daily trajectories (e.g., own school, other schools with recreation facilities, sporting activities) while others afford a higher proportion of active transport trips (e.g., public parks, public transportation services, friends’ houses). The identification of common destinations to which adolescents travel is not only relevant for the development of targeted interventions, but because the importance of these destinations may vary for other population groups who are also exposed to that environment. For example, a built environment change might be more suited or have more influence on one population group compared to another, but understanding these relationships can be a methodological challenge. The location-based methodology performed in this study closely resembles the softGIS methodology developed by Kyttä and colleagues [49,50] which has led to a better understanding of children’s meaningful places from their own perspective. These approaches allow the collection of geographically referenced ‘soft’ information which takes into consideration the individual’s experiential knowledge. When combined with traditional GIS data layers, this type of information has been a welcome addition to evidence-based planning [51,52]. Our results also demonstrate that destinations closer to home (i.e., public parks), have a higher proportion of active transport trips, which is consistent with current literature; distance is one of the strongest predictors of active transportation in young people [53,54]. Being allowed to travel without adult supervision was also associated with greater active travel, alluding to the importance of independent mobility in adolescent populations. Although parental willingness for independent travel is affected by crime and other safety factors [53], parents may be prone to allow greater independent mobility when destinations are closer to home, which translates into greater active travel.

The results presented in Table 4 show the perceived neighbourhood is not uniform in all directions, which highlights the limitation of circular buffers which are derived from fixed radial distances around the home [4]. These data also suggest that the distance from the residence to the edge of the neighbourhood delimitation is not the only variable to consider, but also the shape and positioning of the neighbourhood around the home. Network buffers can somewhat overcome this problem by accounting for environmental barriers and hazards along the street network, yet on average, 20% of the 1 km network buffer area fell outside the perceived neighbourhood. Although the chosen buffer size may have contributed to this discrepancy, network buffers cannot account for destination preferences or the spatial distribution of local resources and amenities around the home, which likely influence the shape of perceived or experienced neighbourhoods. Table 4 also demonstrates that measures of walkability differ between each of the neighbourhood delimitations, suggesting the presence of the modifiable areal unit problem (MAUP); these environmental variables are sensitive to the spatial unit that is applied to the aggregation of these data. It has been demonstrated previously that the association between various environmental attributes and adolescent’s physical activity was dependant on weather a 400 m or 1 mile buffer around the home was used as a spatial aggregation unit (i.e. the neighbourhood) [55]. More recently, differences in built environment variables calculated within six different geographic representations of a neighbourhood highlighted the presence of scaling and zoning effects across these different neighbourhood delimitations [56]. It has been suggested that a strong behavioural justification is needed when deciding how the neighbourhood construct is represented spatially [57]. The interaction between an individual and their environment must be taken into account, yet investigators commonly take a ‘one spatial definition fits all’ approach to defining study subjects neighbourhoods, in which a single neighbourhood definition is applied to all participants regardless of age, gender, location and mobility patterns. A recent study found that adolescent’s self-identified neighbourhoods were not significantly different in area from census-defined neighbourhoods, but the self-identified neighbourhoods were shown to better capture environments where adolescents spent their time and engaged in MVPA [36], which suggests perceived spaces may be a better representation of an individual’s experienced space.

More than half of all geolocated destinations (58%) were beyond the bounds of the perceived neighbourhood. The reality is many adolescents are not restricted to their neighbourhoods during daily living, and are able to use passive methods of transportation to access destinations far beyond their neighbourhood limits. The perceived neighbourhood delimitation might closely represent areas travelled to on foot; an individual may feel a higher level of attachment to that area as these environments are more likely to be experienced intimately. On average, the perceived neighbourhood represented just over a quarter of the total activity space area. These factors support the use of activity space measures that account for the full extent of places visited instead of relying solely on restrictive definitions of place (such as the residential neighbourhood) which could lead to incorrect estimates of exposure [5]. Precise measures of exposure allow for a more robust and revised estimate of the true magnitude of association between behavioral and environmental variables. Improving exposure and outcome variable precision may significantly reduce the chances of a type 2 error; reporting there isn’t an effect when in reality there is.

### Limitations and future applications

Although the use of activity spaces have been proposed as a step forward in this field [5], the spatial area within convex activity-space polygons may contain environments to which the individual is not exposed [16]. Buffering individual destinations and routes between these destinations (buffer size dependant on location and mode of travel) may more closely reflect true exposure [18,58]. The development of domain specific activity spaces (e.g., green space or foodscape exposure) [59] or travel mode activity spaces (e.g., active transport activity space) by circumscribing destinations and routes that are associated with that domain, or travelled to by that mode, will help to isolate environmental effects on the variable of interest. Although VERITAS can obtain retrospective data over extended periods, the data is essentially subjective in nature, and lacks the temporal sequence of events that can be obtained from GPS receivers [16]. Thus, the collective use of activity destination questionnaires and GPS receivers is warranted, as they provide complementary information, leading to more accurate estimates of daily mobility and environmental exposure.

## Conclusions

In summary, the use of activity destination questionnaires with integrated mapping components, such as VERITAS-BEANZ, may help to overcome the shortcomings of previous studies, and are a practical and effective means for attaining geographic information for assessing daily mobility and perceived spaces in adolescent groups.
